# Evolution and distribution of RNA polymerase II regulatory sites from RNA polymerase III dependant mobile Alu elements

**DOI:** 10.1186/1471-2148-4-37

**Published:** 2004-10-04

**Authors:** Ravi Shankar, Deepak Grover, Samir K Brahmachari, Mitali Mukerji

**Affiliations:** 1 Functional Genomics Unit, Institute of Genomics and Integrative Biology (IGIB), CSIR, Mall Road, Delhi 110007, India

## Abstract

**Background:**

The primate-specific Alu elements, which originated 65 million years ago, exist in over a million copies in the human genome. These elements have been involved in genome shuffling and various diseases not only through retrotransposition but also through large scale Alu-Alu mediated recombination. Only a few subfamilies of Alus are currently retropositionally active and show insertion/deletion polymorphisms with associated phenotypes. Retroposition occurs by means of RNA intermediates synthesised by a RNA polymerase III promoter residing in the A-Box and B-Box in these elements. Alus have also been shown to harbour a number of transcription factor binding sites, as well as hormone responsive elements. The distribution of Alus has been shown to be non-random in the human genome and these elements are increasingly being implicated in diverse functions such as transcription, translation, response to stress, nucleosome positioning and imprinting.

**Results:**

We conducted a retrospective analysis of putative functional sites, such as the RNA pol III promoter elements, pol II regulatory elements like hormone responsive elements and ligand-activated receptor binding sites, in Alus of various evolutionary ages. We observe a progressive loss of the RNA pol III transcriptional potential with concomitant accumulation of RNA pol II regulatory sites. We also observe a significant over-representation of Alus harboring these sites in promoter regions of signaling and metabolism genes of chromosome 22, when compared to genes of information pathway components, structural and transport proteins. This difference is not so significant between functional categories in the intronic regions of the same genes.

**Conclusions:**

Our study clearly suggests that Alu elements, through retrotransposition, could distribute functional and regulatable promoter elements, which in the course of subsequent selection might be stabilized in the genome. Exaptation of regulatory elements in the preexisting genes through Alus could thus have contributed to evolution of novel regulatory networks in the primate genomes. With such a wide spectrum of regulatory sites present in Alus, it also becomes imperative to screen for variations in these sites in candidate genes, which are otherwise repeat-masked in studies pertaining to identification of predisposition markers.

## Background

In the post genome sequence era, repetitive sequences, erstwhile considered junk and devoid of function, are increasingly being implicated in many cellular functions, genome organization and diseases [1-8]. Alu repeats, which belong to SINE (short interspersed nucleotide elements) family of repetitive sequences, are present exclusively in the primate genomes. These elements which are ~300 bps in length have originated from the 7SL RNA gene and comprise of two similar, but not identical subunits [9-12]. Each element contains a bipartite promoter for RNA polymerase III, a poly (A) tract located between the monomers, a 3'-terminal poly(A) tract, a number of CpG dinucleotides, and is flanked by short direct repeats [13,14]. Based on certain diagnostic site mutations, they have been broadly classified into three subfamilies: Old (Alu Js), Middle (Alu S) and the Youngest (Alu Ys) [15,16]. Further, some of the Alu Y sequences are very new and exhibit polymorphisms, indicating that they have recently undergone retropositioning process [17].

Alus have been shown to harbor a number of regulatory sites like hormone response element (HRE), and a couple of ligand activated transcription factor binding sites [18-24]. These sites regulate the expression of downstream genes, in some cases in a temporal or tissue specific manner. Most of the regulatory sites in Alus have been reported during the course of characterization of specific genes [25-32]. Besides, the intrinsic A-Box and B-Box RNA polymerase III (RNA pol III) sequences and the recombinogenic sites present in these elements are involved in retrotranspositional and recombination process [10].

Alus originally demonstrated to have non uniform distribution on the chromosomes through banding studies [33,34] have been recently substantiated by genome sequence analysis [35]. It has been observed that that Alus not only show a non- random pattern of distribution in the human chromosomes but also varying densities within genes. Additionally, in a genome wide expression analysis, co-variation of expression of gene pairs has been attributed to sequence similarity metric in the upstream region of promoter predominantly contributed by Alu repeats present in these regions [36]. These effects of Alu have been shown to be completely independent of the effects of isochoric (GC) composition on Alu density as well as gene expression [34-36].

Identification and analysis of various permutations and combinations of these regulatory elements in otherwise conserved repetitive Alus are mostly excluded from genetic analysis. Since, Alus occupy a tenth of the human genome, it is imperative to identify those, which might assume function in the proper context. Our primary aim in this analysis is to find out if any bias exists in the distribution of transcriptional regulatory sites in Alus of various evolutionary ages and their distribution with respect to the functional classes of genes.

## Results and Discussion

### Distribution of functional sites in Alus is position specific

**Figure 1:**
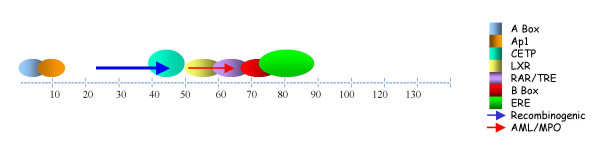
**Representation of regulatory sites on Alu elements. **500 representative Alu sequences each of distinct evolutionary ages were selected for identification of most probable regulatory sites. 126 polymorphic Alus (POLY) from younger subfamilies which show insertion – deletion polymorphisms were also analysed. Sites were identified using local alignment based program as well as by probabilistic modelling approach. These sites are positionally conserved in all subfamilies.

As a first step toward examining the role of these regulatory sites, we mapped their most probable positions on Alus, using in house developed algorithms (Figure 1). This was carried out on 500 Alus, each of Alu Jo, Alu Jb, Alu Sx, Alu Sc, Alu Yb8 and Alu Y subfamilies. The classification of these evolutionarily distinct subfamilies are based on diagnostic sites [15,16,37,38]. Besides, members of the most recent and retropositionally active and polymorphic Alus were also included in the analysis [39,40]. Though the polymorphic Alus belong to Alu Y subfamily, these were treated as a separate category since insertion/deletion of these Alus have been associated with many phenotypes/diseases [2]. The regulatory sites show positional conservation across all subfamilies in which they are represented (Table 1). However, these sites are distinct from the diagnostic sites, which are used for classifying Alus, which suggests that they have not arisen randomly in different subfamilies.

**Table 1:** Position of sites analysed in Alu repeats in various subfamilies.

Family	A-box	B-box	AML	MPO	CETP	Rec	AP1	ERE	RARE	TRE	nCaRE	LXR
*Jb*	5	76	48	48	47	22	13/221	80	57–76	-67	289	
*Jo*	5	76	48	48	47	22	13/221	80	66	-67	289	224–240
*Sx*	5	76	48	48	47	22	13	80	60	-67	289	237–250
*Sc*	5	76	48	48	47	22	13/267	80	68	-67	289	
*Y*	5	76	48	48	47	22		80		-67	289	
*Yb8*	5	76	48	48	47	22	13/270	80	60–66	-67	289	230–240
*POLY*	5	76	48	48	47	22	13/267	80	60	-67	289	

### Evolution of regulatory sites is biased and clustered in Alus

**Figure 2:**
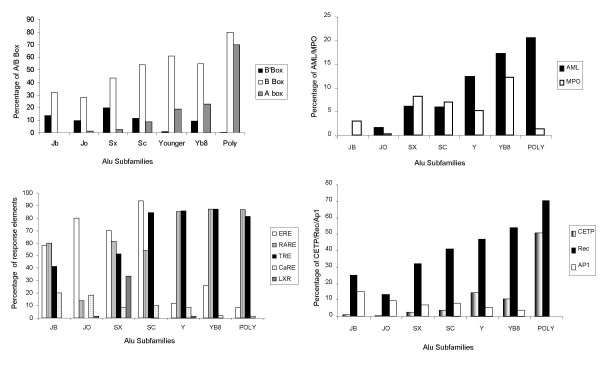
**Distribution of regulatory sites in various Alu subfamilies as well as polymorphic Alus. **On the X-axis Alus of different evolutionary ages as well as polymorphic Alus (POLY) are represented. On the Y-axis the percentage of elements carrying these sites in various subfamilies is indicated.

Nearly all the analyzed regulatory sites for RNA polymerase II (RNA pol II) are distributed in the region between A- Box and B-Box with more clustering near the B-Box region (Figure 1). There is an evolutionary age specific loss / gain of these sites in various subfamilies leading to a bias in their distribution (Figure 2). Newly transposing Alus have methylated CpG sites, which are prone to transition. Many sites seem to have evolved as a consequence of these transitions. The regulatory elements are most abundant in the middle subfamilies and least represented in the younger Alus. Some sites like AP1, ERE, nCARE are present in older and middle Alus but rarely so in the younger as well as polymorphic Alus. An opposite trend is observed for CETP, wherein the highest density is observed in the younger active and polymorphic Alus. RARE and TRE sites are retained in all subfamilies whereas LXR is specific to only middle Alu subfamilies (Figure 2). It is curious, nCARE which is also present in the 7sl RNA, the progenitor of Alus, is not equally represented in all Alus and has higher density in the older Alus and middle and is very poorly represented in the younger subfamilies.

### Evolution from retropositionally active to transcriptionally active Alu elements

Majority of Alu retroposition has ceased at least 30 million years ago and only a few Alu subfamilies are still active [15,17,41]. Transcription of Alus is a prerequisite for retrotransposition and there is regulation not only during transcription initiation but also at the level of stability of transcripts [42]. Alu elements are transcribed by RNA pol III which are composed of two properly spaced conserved sequence motifs, an upstream element named the A-Box and a downstream element called the B Box which are essential for efficient transcription. Deletion of the Box B sequences within the Alu repeat completely abolishes the transcriptional activity. In the absence of box A sequences even though there is a reduction in efficiency of transcription by 10 to 20 fold, B-Box sequence is still capable of initiating transcription 70 bps upstream [43,44]. An intact A Box is therefore a critical determinant for RNA pol III retropositional activity. Besides, it has been shown by *in vitro *as well as in *vivo *studies in the 'B' Box that 'G' and 'T' residues at the 1^st ^and 3^rd ^positions respectively are very critical for it's functioning [45]. Our analysis on the distribution of these promoter elements show that the polymorphic Alu sequences have the highest density of A Box (70%) and is almost absent in older subfamilies (Figure 2). Since the younger Alus are considered to be transcriptionally more active, this fits in well with the loss of this site in the course of evolution due to accumulation of mutations. The B Box motif with the sequence G(A/T)T(C/T)RANNC shows a similar trend as the A Box. Interestingly, a fraction of older Alu subfamily still retains the B-Box sequence. However, 'A' residue at the second position which has not been shown to be critical for transcription is a diagnostic nucleotide [39] for the younger subfamilies. This could result in the increased proportion for B-Box in the younger families. We observe a very curious distribution of the B Box motif if we consider the sequence GTT(C/T)GAGAC (B'Box in Figure 2) wherein we restrict the pattern to the experimentally validated sequence. Alu Sx and Alu Sc have the highest density match with this pattern, followed by the older subfamilies and it is present in only < 2% frequency in AluY and polymorphic Alus. The "C" at the 4^th ^position in this case is mutated to "T" in the older families. The Yb8 family that has been reported to be transcriptionally and retropositionally active amongst the younger subfamilies, retains the B'-Box element in a significant fraction. This suggests that even though retropositionally competent younger Alus are hypothesized to be transcriptionally active, only a minority retains consensus B'-Box. It is possible that the enhancing activity of the A Box is sufficient to drive transcription from the weaker B'- Box in the younger subfamilies. Our findings corroborates well with an earlier study in which presence of all subfamilies in the RNA polymerase III driven Alu transcript pool was reported [46]. Additionally, it was also observed that though there was a quantitative bias towards younger subfamilies and younger members of these subfamilies (based on their relative presence in the transcript compared to their abundance in the genome), there was a preferential expression of the middle subfamilies relative to the most active subfamilies. Our observations, therefore, further rules out the hypothesis that transcription may be biased only towards retropositionally active subfamilies of Alu elements. This could be the reason why only a fraction of younger Alus is currently retrotranspositionally active. The presence and retention of B-Box coupled with near absence of A Box in the Alu Sx and AluSc families suggests basal level of transcription from these elements which could be enhanced through binding of other regulatory proteins under certain conditions such as stress [47]. Additionally, with evidence of presence of naturally occurring Alu antisense as well as edited Alu transcripts [48,49], transcribing Alus could play a major role in yet unknown biological processes.

### Exaptation of Alus in the transcriptional regulatory repertoire

Alus have been demonstrated to exert effects at transcription, post-transcription as well as at the translation level. In an earlier study on complete chromosomes 21 and 22, we have demonstrated that the Alu elements are clustered in genes of signaling, metabolic and transport proteins and rarely present in the structural and information proteins [50]. This clustering bias was found to be irrespective of genomic location, GC content, length of genes or intronic content. To further address whether the Alus harboring transcriptional regulatory sites also show a selective distribution and thereby exert effects on transcription, we analyzed their distribution in the genes of various functional categories of chromosome 22. Two different datasets 1) Promoter region Alus and 2) Intronic region Alus, harboring regulatory sites were analyzed. The promoter region Alus of genes involved in metabolism, signaling were significantly rich in regulatory sites compared to those of information, structure and transport (F value = 4.86, df = 4, 40, p-value < 0.0027). In the intronic regions, distinction in their distribution with respect to functional categories was not so significant though the intronic regions also harboured Alus containing regulatory sites (F value = 2.92, df = 4,40, p-value = 0.032). Since the genes of the signaling and metabolic pathway are more subject to regulation by cellular cues like hormonal triggers, this observation is significant. Most of the Alus in the promoters belong to the middle Alu S families and rarely Younger Alus are present. Since younger Alus also harbour few regulatory sites and actively retropose, it is possible that there is a negative selection against their insertion in the promoter sites of genes of information pathways and structural proteins [see the supplementary data].

### Alu movements and aberrant gene expression

Gene inversions, duplications and formation of pseudogenes have been extensively reported to be mediated both through retrotransposition as well as recombination of Alus. This, in many cases, has also been associated with aberrant gene expression. For instance, presence of AML sites in an Alu upstream of MPO gene, has been first demonstrated to be associated with Acute Myelocytic Leukemia [20]. This is due to the presence of a strong SP1 site within AML which leads to over expression of MPO gene. AML sites are most abundant in younger and polymorphic Alus and a single base pair transition results in MPO site, present predominantly in the members of older subfamilies. In the case of polymorphic Alus, many sequences that do not show 100% conservation of AML site still retain the SP1 site. Interestingly, the core recombinogenic site is also most predominant in younger and polymorphic Alus. The presence of recombinogenic sites in polymorphic Alus, could therefore not only contribute to genome shuffling but also serve to distribute ectopic sites such as AML through retrotransposition and recombination (Figure 2).

### Regulatory region distribution through Alu expansion

**Figure 3:**
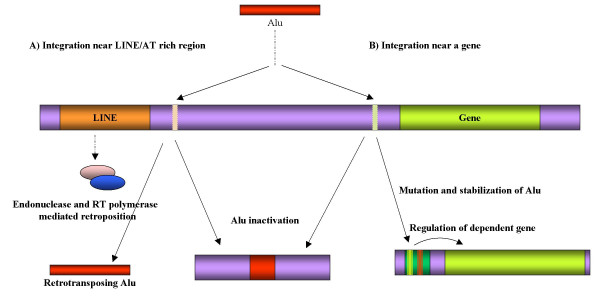
**Alu expansion and evolution of regulatory sites. **With the help of LINEs, Alu may keep on retro-transposing or may get inactive/negatively selected. Alternatively, it may integrate upstream of a gene, accumulate mutations, evolve RNA pol II regulatory sites, get stabilized and control gene expression. This is supported by the presence of sparse regulatory sites, unhindered A box, recombinogenic sites initially in the younger and active Alus and its accumulation in older Alu subfamilies as well as significant presence of Alus harbouring regulatory sites in the promoter encompassing regions of the genes of signaling and metabolic pathways.

Analysis of regulatory sites within Alus suggests that a polymorphic Alu has the potential to transpose and recombine which allows it to integrate at random sites in the genome. They also harbour potential regulatory sites, which could evolve to become accessory sites for RNA pol II transcription as revealed by their clustering in older subfamilies. Further, the Alu sequence due to acquisition of novel functions could form a part of the transcription repertoire involved in the regulation of the downstream /associated genes and create novel regulatory networks (Figure 3). These results also corroborate with the hypothesis of evolution of transposable elements of Kidwell [51] wherein they had proposed a 3 stage life cycle of class II Transposable elements:- invasion and amplification followed by mutations and maturity and finally senescence and fading. In the case of Alu, instead of fading, they could also evolve to become members of host regulatory machinery.

## Conclusions

Comparison of sequences in the regulatory regions of many homologous genes in human have shown accumulation of Alus, not only post divergence from non-human primates but also during primate evolution [52]. Perhaps, recruitment of cis regulatory elements responsive to cellular cues through Alu elements could result in altered spatial and temporal transcription of genes as well as create novel metabolic and signaling networks. These might contribute to the observable physiological complexity in human and primates [53]. Additionally, the underlying events which would be defining event of speciation of human from chimpanzee (with which it shares nearly 99% homology at coding level) still eludes identification and might to some extent reside in such genomic elements. These issues can now be addressed through comparison of these sites in human and chimpanzee.

Currently, Alus are repeat-masked in all studies pertaining to identification of predisposition markers in complex disorders. With such wide spectrum of nuclear receptors, which play a major role in maintaining normal physiological state and affect as diverse processes as development, reproduction, general metabolism, residing in Alus, it therefore becomes imperative to screen for variations in these sites. This might have important consequences in the candidate genes for those complex diseases that are triggered in response to hormonal imbalances as well as other environmental cues.

## Methods

126 polymorphic Alu sequences cited in literature [39,40] were retrieved using NCBI BLAST and Repeat Masker software[54,55]. The analysis was carried out on Alu repeats of human chromosome 22. A randomly selected representative set of approximately 500 Alu sequences, each of distinct evolutionary ages, Alu Jb, Alu Jo, Alu Sx, Alu Sc, Alu Yb8 and Alu Y were used for the analysis. Sequences were retrieved from Sanger Institute Home Page, June 2001 release [56]. Besides, Alus were also analyzed within 5000 base pairs upstream of genes of chromosome 22 in the regulatory regions encompassing promoter sequences as well as inside their intronic regions.

### Collection of biologically active sites

Information about the regulatory sites and their sequences was collected from various literature sources (Table 2). Characteristic features of the sites are given below. We selected those regulatory sites, which have been shown to have function in the Alu elements. The A Box and B Box sequences define the bipartite internal promoters, which bind RNA polymerase III. MPO and AML sites, which are 14 nucleotides differ by an A / G at 5th position of the sequence and transition from G to A at this site converts the MPO allele to AML, resulting in the formation of a strong SP-1 binding site and over expression of the following gene. AP1 sites bind AP-1 transcription factor, which is a dimeric complex that contains members of the JUN, FOS, ATF and MAF protein families. Hormone responsive elements (HRE) are super family of binding sites for ligand activated nuclear hormone receptors for thyroid hormone (TRE), retinoic acid (RARE) and vitamin D, which regulate gene transcription. Estrogen response elements (EREs) are sites for binding of estrogen receptor (ER), a ligand-activated enhancer protein that is a member of the steroid/nuclear receptor super family and transactivates gene expression in response to estradiol. The negative calcium response element type 2 (nCARE) is a regulatory DNA sequence, which inhibits transcription in response to raised extra cellular calcium levels. The nuclear receptors liver X (LXR) is involved in different cell-signaling pathways. CETP site is an orphan receptor site in the Alu in promoter of cholesteryl ester transfer protein (CETP) which plays a key role in reverse cholesterol transport in mediating the transfer of cholesteryl ester from HDL to atherogenic apolipoprotein B-containing lipoproteins.

**Table 2:** Sequences of regulatory elements analysed in Alu repeats.

Site	Sequence
Retinoic acid response element (RARE)	5'(AG)G(GT)TCA 3'
Estrogen Response Element (ERE)	5'(GA)(GA)TCA(CG)(AC)(CG)TGACC 3'
Negative calcium response element (nCARE)	5' TGAGACNNNGTCTCAAAAA 3'
Liver X receptor	5' GACCTNNNNTGATCC 3'
Cholestryl esterase transferase response element (CETP)	5'CCGNGGCGGGC 3'
AP1 site	5' T(GTA)A(GC)TCA 3'
Acute Myelocytic Leukemia (AML) site	5' AGGCGGGTGGATCA 3'
Myelo Peroxidase (MPO) site	5' AGGCAGGTGGATCA 3'
Recombinogenic site	5'CCCTGTAATCCTAGCACTTTGGAGGC 3'
A-Box	5' GGGCGCGGTGGC 3'
B-Box	5' G(A/T)T(C/T)RANNC 3'
B'Box	5' G TT(C/T)GAGAC 3'

Nucleotide sequences in parenthesis indicate alternate nucleotides and have been written in increasing order of their preference.

### Computational methods

Two different programs were written in order to locate the most probable biologically significant regions. A local alignment based program, Xalign, was implemented in C++, Red Hat 7.3 based Linux. This program finds the probable sites by aligning the consensus of regulatory site with the query sequence. Multiple queries with a size upto 600 nucleotides can be taken at a time. Another program, Promotif, was implemented in C++, Red Hat 7.3 based Linux, using the probabilistic modeling approach. It uses the position weight matrix, normalization of the positions with conservation index (Ci Value), and inter-nucleotide dependence in terms of transition matrix to find out the sites. Position weight matrices were generated using Gibbs Motif Sampler, for every site included in the program. The sequences for position weight matrix generation were carefully selected based on the sequence and length reported for each binding site. The final length for search was fixed at the lowest length observed. This provides element specific matrix with lesser chance for the selection on non-RE regions. For the sites analyzed, it had an in built transition matrix, position weight matrix and conservation index. Batch analysis of over a thousand Alu sequences can be performed with this program.

Using the annotated sequences from literature as well as from NCBI web page, training set for the probabilistic model was created. Training was done for approximately 70% sequences and rest of the sequences were taken as test set. Details of the program along with the equations used are available on request.

### Mapping of recently integrated and younger Alus

About 126 recently integrated Alus from younger subfamilies were searched in the human genome using BLASTn at NCBI server and regulatory sites were mapped in these regions using the programs discussed above.

## Association analysis

Alus in the promoter regions and intronic regions of functionally classified genes [50] of chromosome 22 were mapped and pattern of distribution of biologically significant sites were analyzed by ANOVA.

## Authors' contributions

RS developed the algorithms and programs for identifying regulatory and significant regions, carried out the analysis of distribution of these sites in Alu subfamilies, association analysis and drafted the manuscript. DG was involved in chromosome 22 analyses. SKB participated in the design of the study. MM conceived of the study, participated in its design, analysis, coordination and manuscript preparation. All authors read and approved the final manuscript.

## Supplementary Material

Supplementary data The analysis over the promoter and intronic regions has been performed through the data given in the supplementary table file, supplementary table 3_ravishankar et al. Format: .xls. For human chromosome 22, the data contains the accession number, associated Alu family, the respective positions, functional class of the region and further details, for each associated regulatory element found within the Alu repeats in the 5' flanking promoter and intronic regions. The zipped file name is supplementary 1.zip. Details about programs used are on request for academic users. Click here for file
